# Identification and mitigation of errors in hole spin qubit readout

**DOI:** 10.1038/s41928-026-01705-1

**Published:** 2026-09-29

**Authors:** Eoin G. Kelly, Leonardo Massai, Bence Hetényi, Marta Pita-Vidal, Alexei Orekhov, Cornelius Carlsson, Inga Seidler, Konstantinos Tsoukalas, Lisa Sommer, Michele Aldeghi, Stephen W. Bedell, Stephan Paredes, Felix J. Schupp, Matthias Mergenthaler, Andreas Fuhrer, Gian Salis, Patrick Harvey-Collard

**Affiliations:** 1 IBM Research Europe—Zurich, Rüschlikon, Switzerland; 2 IBM Quantum, T.J. Watson Research Center, Yorktown Heights, NY USA

**Keywords:** Electronic and spintronic devices, Quantum information, Qubits, Quantum dots

## Abstract

High-fidelity readout of spin qubits in semiconductor quantum dots can be achieved by combining a radiofrequency charge sensor with spin-to-charge conversion and Pauli spin blockade. However, reaching high readout fidelities with hole spin qubits remains challenging due to site-dependent spin anisotropies and short relaxation times during readout. Here we analyse the multiple error processes that arise during readout using a double-latched scheme in a germanium double-quantum-dot hole spin qubit system. We first investigate the spin-to-charge conversion process as a function of magnetic field orientation and configure the system to adiabatically map the $\left\vert \downarrow \downarrow \right\rangle$ state to the only non-blockaded state. We reveal a strong magnetic field strength dependence of the spin relaxation rates, and minimize it by operating at low fields. We further characterize and mitigate the error processes that arise during the double-latching process. By combining a radiofrequency charge sensor, a double-latching process and optimized magnetic field parameters, we achieve a single-shot, single-shot fidelity of 97.0% for single-qubit state preparation and measurement while maintaining control of both spins under the readout conditions.

## Abstract (web summary)

A double-latched readout scheme in a germanium double-quantum-dot hole spin qubit system provides a single-shot fidelity of 97.0% for single-qubit state preparation and measurement.

## Main

Hole spins in germanium quantum dots (QDs) are a promising spin qubit platform for a variety of reasons: high-quality heterostructures leading to low charge noise and disorder^1,2^, all-electrical manipulation due to strong spin–orbit interaction^3^, operational sweetspots for qubit coherence^4^ and high-fidelity qubit operations^5,6^. Spin readout in these systems is typically performed using Pauli spin blockade (PSB) to encode spin into charge, followed by charge readout using a charge sensor. Measuring the charge sensor via radio-frequency (RF) reflectometry can provide high sensitivity and a large bandwidth^7^. A latched readout technique^8^ has previously been used in germanium^9^ to enhance the readout signal and lifetime of the charge states during the measurement. Furthermore, a double-latching mechanism^10^ for spin qubit readout has recently been shown to increase the lifetime of metastable latched charge states, which are limited by charge co-tunnelling.

However, achieving >99% fidelity readout of hole spins remains a challenge. Such high-fidelity readout has been demonstrated in electron spin qubit systems based on silicon^11–14^, but the highest reported fidelity for holes in germanium lies around 94%^5^. Increasing the readout fidelity will require a detailed understanding of how strong spin–orbit interaction and *g*-tensor anisotropies affect spin-to-charge conversion (StCC) and readout errors.

In this Article, we analyse StCC and the various decay processes that can occur during spin readout in a germanium hole double-QD system with an RF charge sensor. After a brief review of the enhanced latching readout, we first investigate the Landau–Zener physics of passing through two different singlet–triplet anticrossings induced by site-dependent spin anisotropies. We distinguish three different StCC regimes based on diabaticity with respect to these anticrossings and access each in turn by varying preparation and readout ramp times. We then investigate the ramp time required to reach a particular regime as a function of the magnetic field orientation, as well as the errors that arise during StCC. We furthermore consider spin decay in the PSB window and find that spin decay times are increased by operating at low field strengths. The errors resulting from spin and charge decay during latched mapping are also investigated. Finally, leveraging the understanding acquired about each readout process, we demonstrate an average single-qubit and single-shot state preparation and measurement (SPAM) fidelity of 97.0(5)% using double latching, primarily limited by imperfect state preparation and signal-to-noise ratio (SNR).

## Double-latched readout scheme

**Extended Data Fig. 1:**
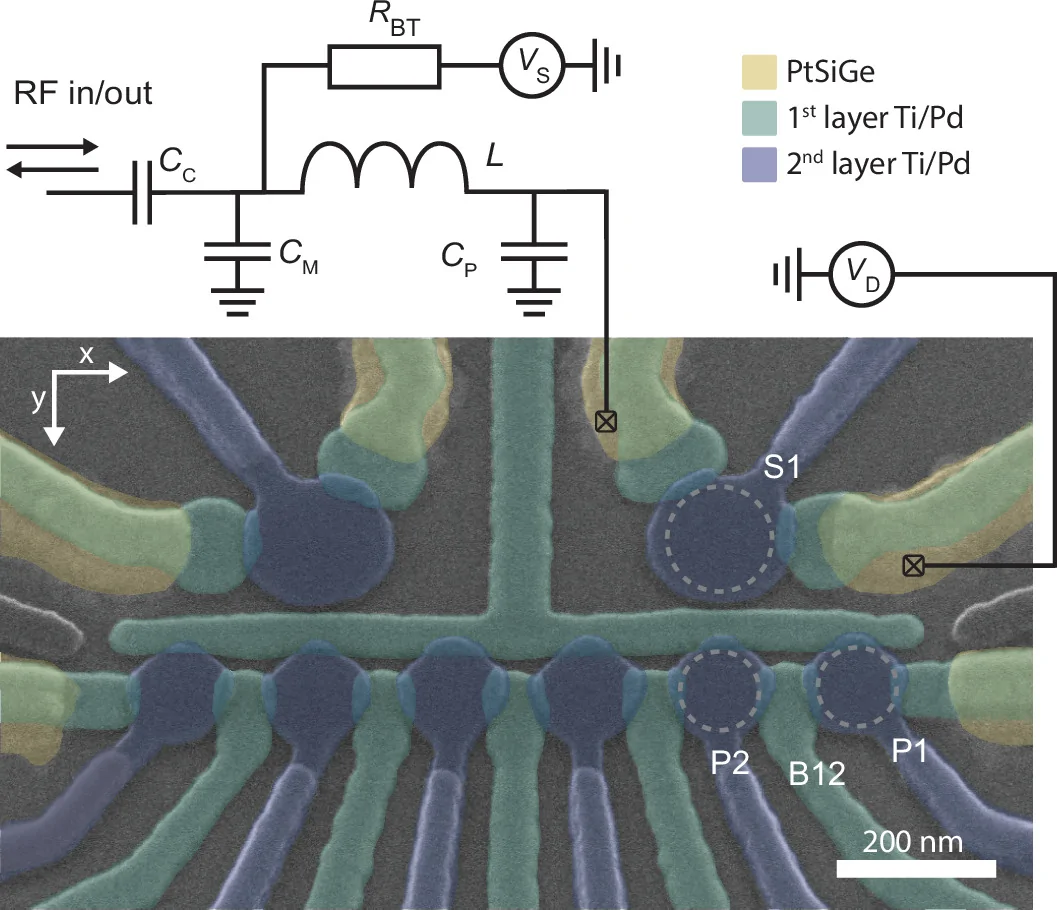
Device and readout circuit. Ge quantum dot (QD) device with RF single-hole transistor, and schematic of the readout circuit (resistors *R*, capacitors *C*, inductors *L*, voltage source *V*).

The device used in this experiment is a linear six QD array with two charge sensors^15^ created on a Ge/SiGe heterostructure containing a strained Ge quantum well. The device details are reported in the ‘Device and experimental setup’ section in Methods and Extended Data Fig. 1. Two single-hole QDs at one end of the array, QD1 and QD2, are formed underneath plunger gates P1 and P2 and are capacitively coupled to a nearby charge sensor. The interdot tunnel coupling between the two QDs is tuned by a barrier gate B12. An inductor-capacitor impedance matching circuit utilizing a niobium nitride (NbN) superconducting nanowire inductor on a separate chip^16,17^ is used to detect changes in impedance of the charge sensor via RF reflectometry^7^.

A grounded screening layer in the first gate layer of the device is used to shield the sensor RF excitation from undesired dissipation in the heterostructure. On the one hand, this ensures signal collection from the charge sensor but on the other hand forces a trade-off with charge sensitivity, which is negatively impacted by the *C*_p_ ≈ 6 pF parasitic capacitance introduced (Supplementary Section 1). The measurement bandwidth could be substantially improved in the future by using a heterostructure with less dissipation, allowing to forego the screening layer.

**Fig. 1:**
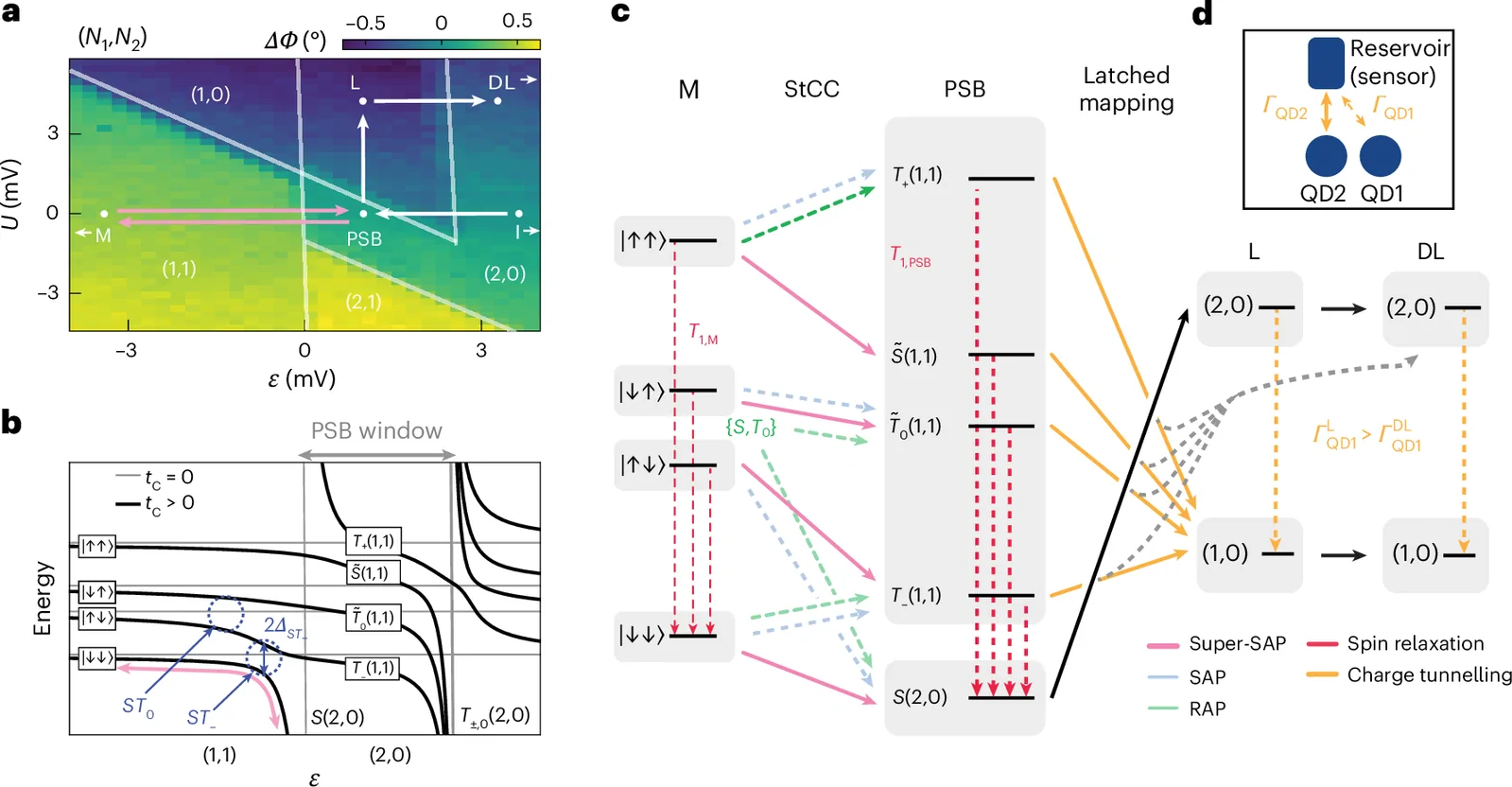
Double-latched readout processes. **a**, Double-QD charge stability diagram where the virtual detuning *ε* and on-site energy *U* are varied, and the reflectometric change in phase Δ*Φ* is plotted. The points and arrows indicate the pulse sequence. Arrows beside labels indicate points outside the scan area. The pulse sequence also involves the barrier gate vB12 (Fig. 4a). The plot is the result of varying the L point after preparing a blocked state at point M (DL point not used). **b**, Simulated two-spin energy spectrum as a function of *ε* with *U* = 0 mV, with details given in Supplementary Section 3. Here, *t*_c_ refers to the interdot tunnel coupling. The polarized spin states *T*_±_(1, 1) hybridize with the singlet *S*(2, 0) due to a combination of *g*-factor anisotropy and spin-flip tunnelling, leading to an anticrossing of size $2{\Delta }_{S{T}_{-}}$. The location of the *ST*_0_ anticrossing (that is, where the exchange energy equals the Zeeman splitting difference) is also indicated. **c**, Summary of the conversion processes involved in the readout. Starting from a qubit state at point M, the arrows indicate possible paths that might lead to the desired outcome (full lines) or errors (dashed lines). The diagram can also be used to understand the contribution of each step to the total readout time, including the time to ramp from M to PSB, the time to wait at L and the charge readout time at DL. Relaxation times *T*_1_ contribute to errors. **d**, A schematic of the connectivity of the double-QD system, where QD-reservoir transition rates denoted by *Γ* must be largely asymmetric. I, initialization point; PSB, point inside the PSB window; M, manipulation point where qubits are operated; L, latched point, where enhanced latched mapping takes place; DL, double-latched point, used to extend the lifetime of the metastable state after mapping.

Due to the short relaxation time of blocked spin states, a double-latched readout scheme^10^ is used in this experiment to enhance the readout signal’s lifetime^8^. The scheme is illustrated in Fig. 1a, where the charge stability diagram around the (1, 1) to (2, 0) charge sectors is plotted along with the pulse sequence. The measured quantity is the change in phase Δ*Φ* of the reflectometry signal. Here (*N*_1_, *N*_2_) represents the hole charge state of QD1 and QD2, respectively. The device is tuned to load holes from the charge sensor as indicated in Fig. 1d, which naturally provides the asymmetry in loading rates required for latched readout. The applied gate voltages are parameterized by the on-site energy *U* and detuning *ε* virtual control parameters, which are linear combinations of virtual gate voltages vP1 and vP2 (‘Virtual gates, detuning and on-site energy’ section in Methods).

**Extended Data Fig. 2:**
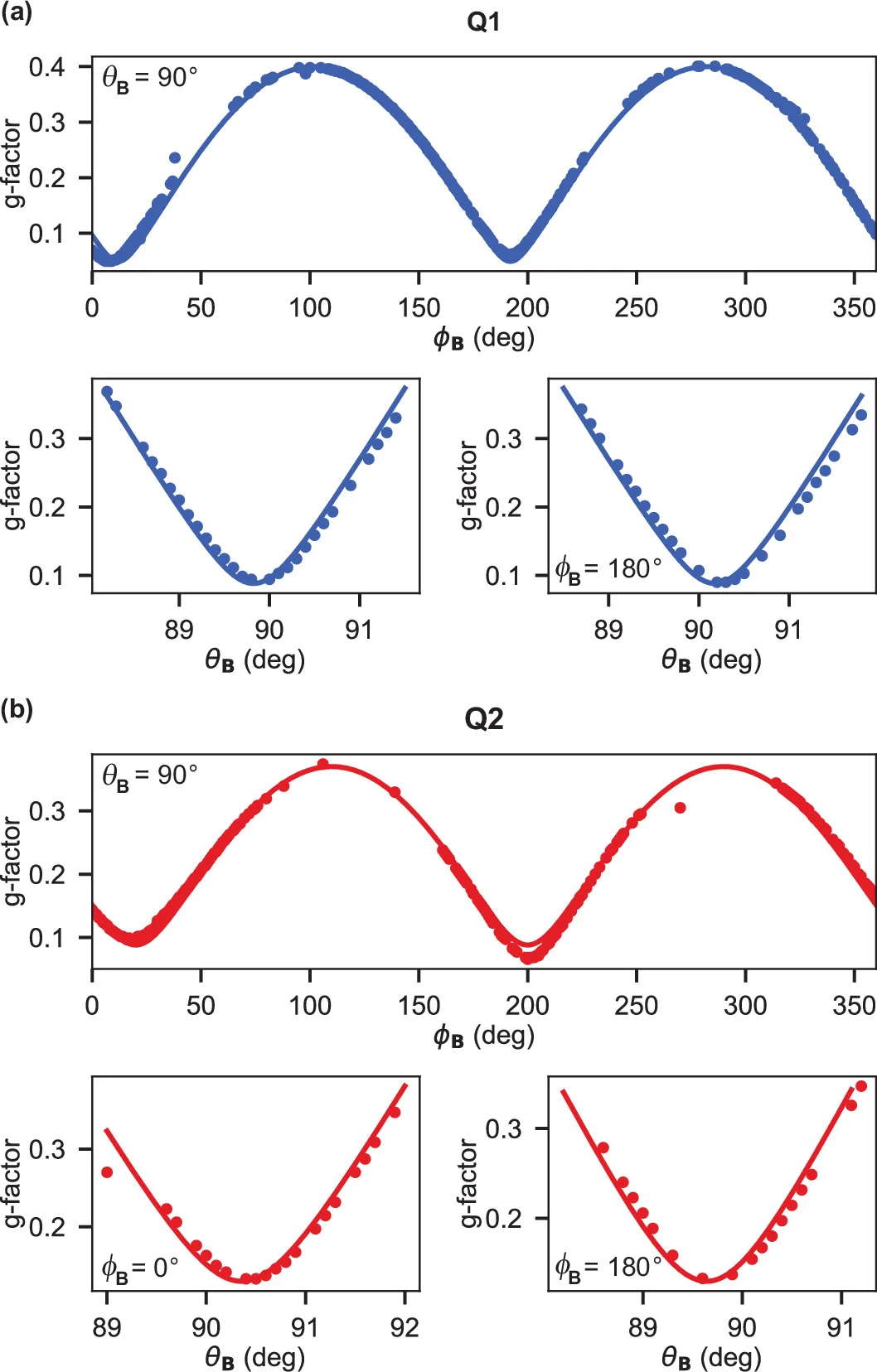
Measured g-factors and their fits (lines). (**a**) Q1 and (**b**) Q2 as a function of magnetic field orientation with *B* = 80 mT.

The complete readout process involves a sequence of steps with many possibilities for errors to occur and is comprehensively described in Fig. 1c. StCC of computational states is first performed by ramping *ε* from the manipulation point M to point PSB within the PSB window. The barrier is also changed to increase the tunnel coupling before this ramp. This maps the states to either the unblocked *S*(2, 0) state or blocked $\{{T}_{-}(1,1),{\widetilde{{T}}}_{0}(1,1),\tilde{S}(1,1),{T}_{+}(1,1)\}$ states at point PSB. Here, the tilde represents the hybridization of *T*_0_(1, 1) and *S*(1, 1) due to the interplay between the *g*-factor difference and exchange inside the PSB window (see the *g*-factors in Extended Data Fig. 2). Furthermore, we take the arrow symbol in the kets such as $\left\vert \downarrow \downarrow \right\rangle ,\left\vert \uparrow \uparrow \right\rangle$ to represent the ground state and excited state indices, respectively, instead of representing the direction of the spin in real space. This is because the spins could point in very different directions in the two QDs.

Depending on the adiabaticity of the ramp with respect to both the *ST*_−_ and *ST*_0_ anticrossings, three different StCC schemes are possible. Here, it is assumed that the ramp is always adiabatic with respect to charge. Super-slow adiabatic passage (super-SAP) is adiabatic with respect to both anticrossings, resulting in a mapping depicted by the pink arrows in Fig. 1c. Slow adiabatic passage (SAP) is adiabatic with respect to the *ST*_0_ anticrossing but diabatic with respect to the ST_−_ anticrossing, resulting in a mapping depicted by the blue arrows^18^. Rapid adiabatic passage (RAP) is diabatic with respect to both anticrossings (Fig. 1c, green arrows). A table summarizing these different regimes is given in the ‘StCC mapping schemes’ section in Methods and Extended Data Table 1.

Operating with super-SAP allows for a state-selective StCC of $\left\vert \downarrow \downarrow \right\rangle$ to *S*(2, 0), with all other computational states being converted to (1, 1) states within the PSB window. We later refer to this generalization of the singlet–triplet readout as the 1 versus 3 readout (‘StCC mapping schemes’ section in Methods). As we will show, this is the optimal StCC regime to use in systems with a large *ST*_−_ anticrossing. Adiabaticity takes care of preserving the weight of the eigenstates during StCC, alleviating the need to monitor the direction of the quantization axis of each QD.

For initialization, the symmetry of the StCC process is leveraged. A singlet *S*(2, 0) state is first prepared by waiting approximately 10 μs at point I, deep in the (2, 0) region. The $\left\vert \downarrow \downarrow \right\rangle$ state is then prepared by adiabatically ramping *ε* through the *ST*_−_ anticrossing as depicted by the pink arrow in Fig. 1b. This ramp is performed with a large exchange energy *J* (and therefore tunnel coupling) to facilitate StCC. However, this exchange couples the two spins too much for independent single-spin operation. Therefore, *J* is then adiabatically reduced to *J*/*h* < 1 MHz using the virtual vB12 barrier gate before qubit operation. Any other computational spin state $\{\left\vert \uparrow \downarrow \right\rangle ,\left\vert \downarrow \uparrow \right\rangle ,\left\vert \uparrow \uparrow \right\rangle \}$ can then be prepared by the application of RF control pulses on gates P1 and P2.

After qubit operation and subsequent StCC, the blocked (1, 1) states can decay rapidly to *S*(2, 0) at the PSB point due to spin–orbit interaction^19^. This limits the readout fidelity if the required measurement time is comparable to the spin decay time. This decay can be avoided by quickly mapping the spin state to a much more stable charge state via a latching process^8^. To enable this, we choose asymmetric tunnel rates *Γ*_QD*i*_ of each dot *i* to the reservoir, such that *Γ*_QD2_ is much faster than the PSB decay rate, whereas *Γ*_QD1_ is much longer than the measurement time. By pulsing from point PSB to the latching point L, the blocked (1, 1) spin state is thereby quickly mapped to (1, 0) with rate *Γ*_QD2_, while the *S*(2, 0) state remains as (2, 0). This scheme avoids spin decay of the (1, 1) spin states. Using a latched readout scheme also has the benefit of enhancing the contrast between the two states because they now differ by a total charge, compared with a change in charge distribution in the normal PSB case^20^.

The locked (2, 0) metastable charge state can now decay to (1, 0) by co-tunnelling. To further increase its lifetime, an extra step to a double-latched point DL positioned at a large detuning is used. This reduces co-tunnelling through the other dot. As will be shown, reading out at DL greatly enhances the lifetime of the metastable (2, 0) charge state and is highly effective in preventing further errors before readout. However, because DL is outside the PSB window, incomplete conversion at latched point L by not waiting long enough there before DL can lead to errors (Fig. 1c, dashed grey lines).

## StCC errors

**Fig. 2:**
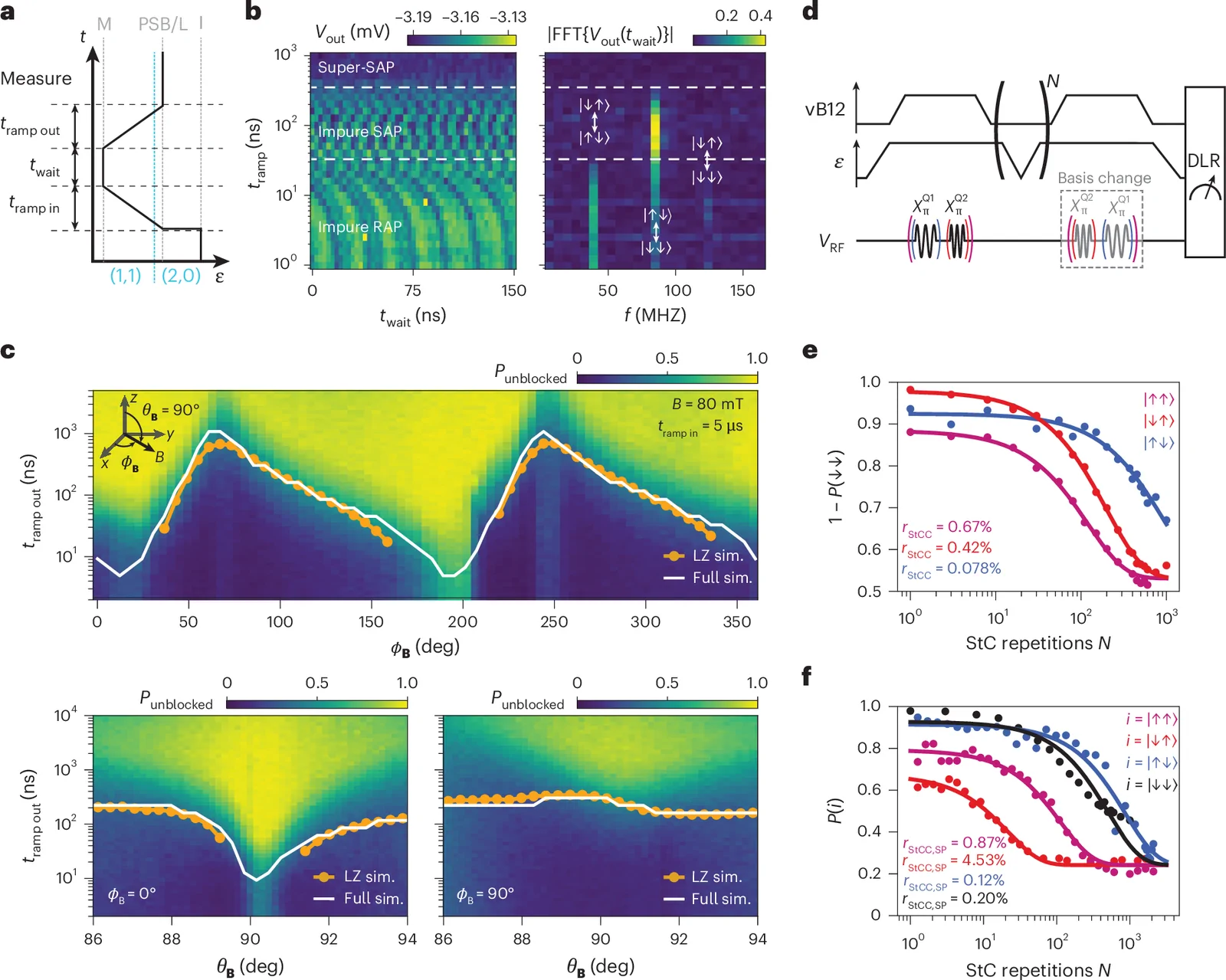
StCC. **a**, Pulsing sequence associated with the investigation of the different StCC regimes. **b**, Variation of *t*_ramp_ = *t*_rampin_ = *t*_rampout_ versus *t*_wait_, together with the fast Fourier transform (FFT) along *t*_wait_ of the reflectometry voltage *V*_out_. Different transition frequencies are revealed in different StCC regimes, namely super-SAP, SAP and RAP. **c**, Variation of *t*_rampout_ for a fixed *t*_rampin_ = 5 μs as a function of magnetic field orientation in three different planes with *B* = 80 mT. *P*_unblocked_ is the fraction of unblocked measurements. The orange and white lines represent the adiabatic-diabatic crossover simulated using the Landau–Zener formula or a time-evolution of the system Hamiltonian, as detailed in the main text. **d**, The pulsing sequence used to determine the StCC errors in the super-SAP regime of **e** and **f**. A specific (1, 1) spin eigenstate in {$\left\vert \uparrow \downarrow \right\rangle ,\left\vert \downarrow \uparrow \right\rangle ,\left\vert \uparrow \uparrow \right\rangle$} is first prepared (blue, red and magenta pulses, respectively), followed by a symmetric StCC process repeated *N* times, before a final StCC step and double-latched readout (DLR). *P*(*i*) represents the fraction of shots where the two spins were assigned the state $\left\vert i\right\rangle$. **e**, Without the final basis change π-pulses *X*_π_, the protocol extracts the StCC error per double ramp *r*_StCC_ (fitted lines) for different input states (legend). This contributes errors in the normal sense of PSB readout. **f**, With the final basis change pulses, the protocol extracts the probability per double ramp *r*_StCC,SP_ that the input state leaks to any other state. This process is not necessarily a readout error if the mixing occurs within the blocked subspace. Both **e** and **f** are measured with $\left({\phi }_{{\bf{B}}},{\theta }_{{\bf{B}}}\right)=\left(19{4}^{\circ },9{0}^{\circ }\right)$. LZ, Landau–Zener; sim., simulation.

We now experimentally study the best StCC regime for state preparation and readout. We first initialize a *S*(2, 0) state and then pulse *ε* from PSB to M. We vary the ramp times to (*t*_rampin_) and from (*t*_rampout_) the M point, together with the wait time (*t*_wait_) at M. The resulting reflectometry signal *V*_out_ is shown in Fig. 2b. We set the magnetic field *B* = 40 mT parallel to the QD array, with equal *t*_ramp_ = *t*_rampin_ = *t*_rampout_ and plot the results in Fig. 2b.

For the shortest *t*_ramp_, we first observe an impure RAP regime (‘StCC mapping schemes’ section in Methods). Coherent oscillations are observed in the readout signal as a function of *t*_wait_ at M (ref. ^21^), as the quick ramp from *S*(2, 0) into the (1, 1) charge region prepares a superposition of eigenstates at M. Applying a fast Fourier transform to the data extracts three different oscillation frequencies, as displayed in Fig. 2b, right. These frequencies correspond to all possible transitions between the $\{\left\vert \downarrow \downarrow \right\rangle ,\left\vert \uparrow \downarrow \right\rangle ,\left\vert \downarrow \uparrow \right\rangle \}$ states.

As *t*_ramp_ is increased, coherent oscillations between $\left\vert \downarrow \downarrow \right\rangle \leftrightarrow \left\vert \uparrow \downarrow \right\rangle$ become dominant. This happens when the ramp rate is completely adiabatic with respect to the *ST*_0_ anticrossing. Superpositions with respect to the *ST*_−_ anticrossing are now dominant (Fig. 2b, impure SAP). As *t*_ramp_ is increased even further, no coherent oscillations are observed, indicating that the super-SAP regime has been achieved. Here, $\left\vert \downarrow \downarrow \right\rangle$ is mapped to *S*(2, 0) by adiabatically traversing the *ST*_−_ anticrossing. The different StCC maps are summarized in Fig. 1c. and the ‘StCC mapping schemes’ section in Methods.

At this field orientation, we find no region without coherent oscillations between the RAP and SAP regimes, as evident from Fig. 2b. In other words, there is no ramp time which is completely adiabatic with respect to the *ST*_0_ anticrossing while being completely diabatic with respect to the *ST*_−_ anticrossing. This is due to the large *ST*_−_ anticrossing. Previously, a variable ramp rate (double ramp)^22^ has been used to traverse the two anticrossings at different rates. In our case, because the regions in detuning where the two anticrossings occur tend to overlap (Fig. 1b), this scheme is not practical. We therefore conclude that in our case, the super-SAP scheme is more suitable. However, when the *ST*_−_ gap is much smaller than the *ST*_0_ gap, often the SAP regime is preferable^23^. Given this possibility to switch between SAP and super-SAP, the time required by the StCC step should not limit the total readout time until the charge detectors can achieve low integration times of order 100 ns to 1 μs.

As optimal points for qubit coherence exist at certain magnetic field orientations due to the highly anisotropic nature of hole *g*-tensors in Ge (ref. ^4^), we investigate the ramp time *t*_ssap_ required to reach the super-SAP regime as a function of field orientation. A method for finding *t*_ssap_ is to prepare $\left\vert \downarrow \downarrow \right\rangle$ by using a fixed, large *t*_ramp in_ and vary *t*_ramp out_, while measuring the resulting unblocked state probability *P*_unblocked_(*t*_ramp out_). Such measurements are reported in Fig. 2c as a function of the azimuthal and polar angles of the magnetic field $\left({\phi }_{{\bf{B}}},{\theta }_{{\bf{B}}}\right)$. Here, *t*_ssap_ is defined such that *P*_unblocked_(*t*_ssap_) = 1 − 1/*e*. This time varies by orders of magnitude depending on the field orientation due to the modulation of the size of the *ST*_−_ anticrossing with field direction. *P*_unblocked_ can be modelled by the Landau–Zener (LZ) probability of adiabatically transitioning through the *ST*_−_ anticrossing in cases where this anticrossing is well defined^24^

1 $${P}_{\mathrm{LZ}}({t}_{\mathrm{ramp}})=1-{\rm{exp}} \left(-\frac{{t}_{\mathrm{ramp}}{(\pi {\varDelta }_{S{T}_{-}})}^{2}}{h\left|({\varepsilon }_{\mathrm{PSB}}-{\varepsilon }_{{\rm{M}}})\frac{{\rm{d}}{E}_{S{T}_{-}}}{{\rm{d}}\varepsilon}\right|}\right).$$

We determine *t*_ssap_ for a specific magnetic field **B** by calculating the energy spectrum as a function of *ε*, and then finding both the size of the anticrossing ${\varDelta }_{{{\rm{ST}}}_{-}}$ and slope $\frac{{\rm{d}}{E}_{S{T}_{-}}}{{\rm{d}}\varepsilon }$ of the *ST*_−_ energy difference versus *ε* around the anticrossing. This requires the determination of the hole *g*-tensors of the two QDs, which are presented in the ‘Measurement of qubit *g*-tensors’ section in Methods and Extended Data Fig. 2 and a spin-flip tunnelling contribution (explained below). The calculated *t*_ssap_ from equation (1) is in agreement with the data for most field orientations (Fig. 2c, orange dots). The Landau–Zener approximation breaks down, however, when the anticrossing is comparable to the qubit energy splitting, leading to no well defined minimum in the *ST*_−_ energy difference.

To model the data for all field angles, we use a time-dependent five-state Hamiltonian to propagate the $\left\vert \downarrow \downarrow \right\rangle$ state as a function of time and determine the probability of $\left\vert \downarrow \downarrow \right\rangle$ evolving into a *S*(2, 0) state for various ramp times. The result of such a simulation is presented as the white lines in Fig. 2c. In addition to knowledge of the qubit *g*-tensors, a spin-flip tunnelling term^25,26^ is required to fit the data (Supplementary Section 2). The fit allows the extraction of this spin-flip tunnelling term. We find the smallest *t*_ssap_ at $\left({\phi }_{{\bf{B}}},{\theta }_{{\bf{B}}}\right)=\left({194}^{\circ },{90}^{\circ }\right)$ and operate at this orientation to minimize spin decay errors during the passage through the *ST*_−_ anticrossing. We find empirically that the range of in-plane magnetic fields where super-SAP can be achieved is quite large in the Ge devices we have worked with. If one sets an arbitrary threshold of a 1 μs adiabatic ramp, nearly all angles except two spots spanning ~10° to 20° are suitable (Fig. 2c).

To measure the errors that arise during the StCC ramps, we repeat a symmetric StCC step an increasing number of times *N* before readout (Fig. 2d). All four two-qubit basis states are prepared in turn by super-SAP preparation of $\left\vert \downarrow \downarrow \right\rangle$ with *t*_rampin_ = 120 ns, followed by the application of single spin-flip pulses. After this input state preparation, a super-SAP back-and-forth ramp (M → PSB → M) is repeated *N* times. Following this, the population of the specific input state $\left\vert i\right\rangle$ can be optionally mapped to $\left\vert \downarrow \downarrow \right\rangle$ by further qubit control pulses making the readout selective to that state. We refer to this hereafter as a readout basis change step. Lastly, a final StCC mapping followed by double-latched readout is performed.

We define the StCC error *r*_StCC_ as the probability of not detecting the signal of the prepared state after one back-and-forth ramp. In the absence of a readout basis change step (Fig. 2e), we obtain an average *r*_StCC_ for the three blocked spin states of 0.39% per repetition by fitting the data to an exponential decay $\sim \exp (-N{r}_{{\rm{StCC}}})$ (with scale and offset, valid when *r*_StCC_ ≪ 1). With a readout basis change step, this measurement extracts the state-preserving StCC error *r*_StCC,SP_ of state *i* evolving to any other state than itself. We observe an average *r*_StCC,SP_ of 1.43%, limited primarily by the high *r*_StCC,SP_ of $\left\vert \downarrow \uparrow \right\rangle$ (Fig. 2f). Note that errors extracted in this way would not lead to a measurement error when the leakage occurs between the blocked states. It is therefore a more stringent measure that is similar to a quasi-non-demolition measurement of the $\left\vert \downarrow \downarrow \right\rangle$ versus $\{\left\vert \downarrow \uparrow \right\rangle ,\left\vert \uparrow \downarrow \right\rangle ,\left\vert \uparrow \uparrow \right\rangle \}$ two-spin subspaces, in the sense that it potentially disregards phase coherence but preserves eigenstate probabilities^27^.

## Readout decay processes

As outlined in Fig. 1c, there are several decay processes that affect readout fidelity. Decay before reaching the PSB point is measured with the method presented in the previous section. We now focus on spin decay at the PSB point and charge mapping errors that occur during latched mapping.

**Fig. 3:**
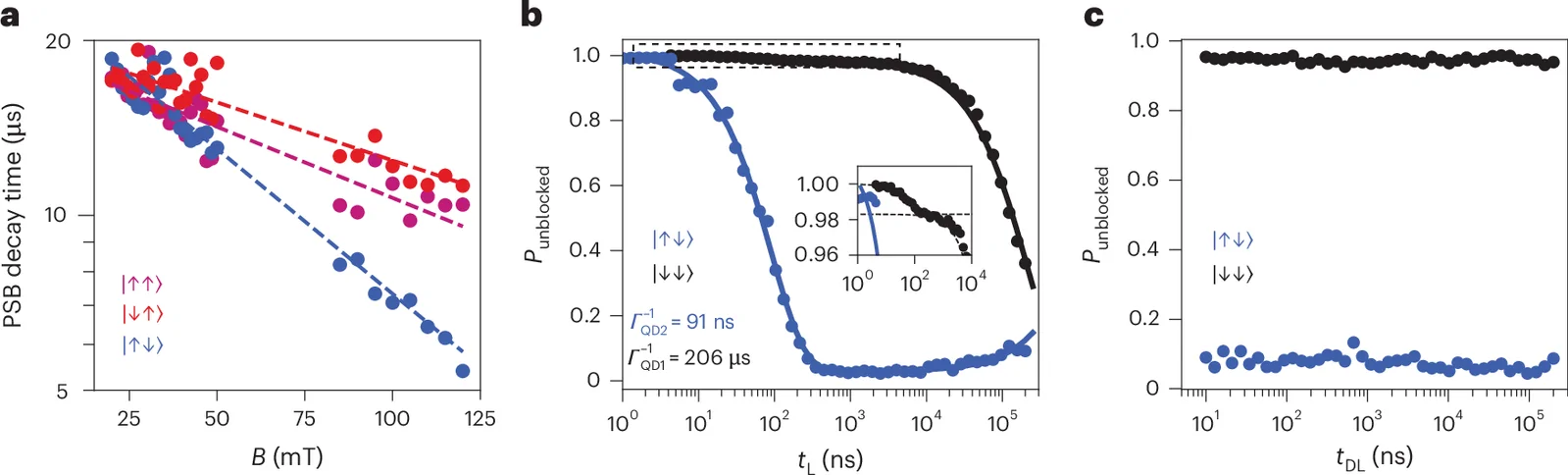
Readout decay processes. **a**, Characteristic spin decay times ${\Gamma }_{\mathrm{PSB}}^{-1}$ at the PSB point of the three excited spin eigenstates as a function of magnetic field amplitude *B*. Dashed lines are exponential decay fits to the data. See the ‘PSB decay versus in-plane magnetic field orientation’ section in Methods for the angular dependence. **b**, Probability of measuring the unblocked (2, 0) charge state *P*_unblocked_ as a function of time *t*_L_ at the L point for two different input states (blue and black). The inset shows the two-step exponential decay that occurs for $\left\vert \downarrow \downarrow \right\rangle$ within the dashed box. Lines are two-step exponential decay fits to the data. **c**, *P*_unblocked_ as a function of wait time *t*_DL_ at point DL before measurement for two different spin states (*t*_L_ = 2 μs).

**Extended Data Fig. 3:**
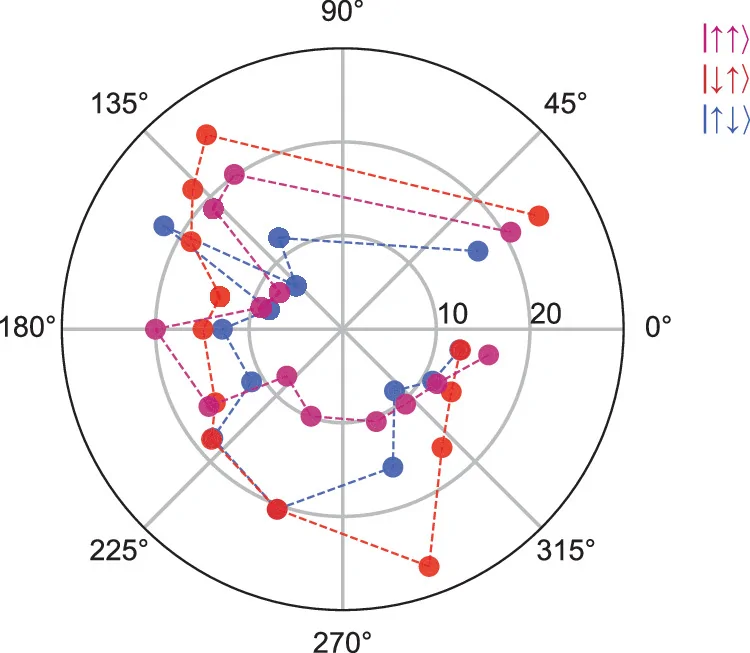
Angular dependence of decay times. Measured decay times (in μs) at the PSB point for $\{\left\vert \uparrow \downarrow \right\rangle ,\left\vert \downarrow \uparrow \right\rangle ,\left\vert \uparrow \uparrow \right\rangle \}$ as a function of in-plane magnetic field angle *φ*_**B**_ with *θ*_**B**_ = 90 for constant *B* = 80 mT.

Spin decay times are extracted for the blocked spin states at the PSB point as a function of *B* with (*ϕ*_**B**_, *θ*_**B**_) = (196°, 90°), and plotted in Fig. 3a. The results reveal an approximately exponential decrease of the decay times for all states with increasing *B*, with all of them starting around 20 μs at the lowest *B*. This exponential decrease contrasts with the power law behaviour previously predicted and measured for spin qubits^28–30^. Lower lifetimes with increasing *B* suggests that operating at low *B* is beneficial not only for qubit coherence, as previously reported for planar Ge (refs. ^4,5^) but also for readout. Furthermore, the decay times of the polarized and antipolarized states do not differ much from each other (less than an order of magnitude). This may be due to a long relaxation time of $\tilde{S}(1,1)$ to *S*(2, 0) at the PSB point^31^. This leads to the singlet–triplet-mode readout observed here which, combined with single-spin rotations, can selectively read any one state in the two-spin Hilbert space from the other three. By contrast, the parity-mode readout observed in other works^12,14^ is never observed under our conditions, even for different in-plane field orientations (‘PSB decay versus in-plane magnetic field orientation’ section in Methods; Extended Data Fig. 3) and strengths (Fig. 3a), and tunnel couplings are ~1 GHz (±factor of ~5).

For latched mapping, the time spent at the PSB point can be substantially reduced, but the decay time at the PSB point ${\Gamma }_{\mathrm{PSB}}^{-1}$ can still be a prevalent source of error as it is in competition with the fast but finite tunnel rate to the reservoir. We refer to this competition between paths as ‘the fork’. The probability of this decay occurring before latching is given by *Γ*_PSB_/(*Γ*_PSB_ + *Γ*_QD2_) (ref. ^8^). *Γ*_QD2_ is found here by measuring *P*_unblocked_ after preparing $\left\vert \uparrow \downarrow \right\rangle$ (Fig. 3b) as a function of *t*_L_, the wait time at L. An exponential decay to (1, 0) as a function of *t*_L_ occurs with a characteristic time ${\Gamma }_{\mathrm{QD}2}^{-1}=91\,\mathrm{ns}$. In our case, the error arising from the fork is estimated to be around 0.46%, using the PSB decay time of ${\Gamma }_{{PSB}}^{-{1}}=20\,\upmu {\rm{s}}$ observed for the three blocked states at 20 mT (Fig. 3a). It is worth pointing out that given these short PSB decay times, any sensor would need to be similarly fast (91-ns measurement time) to reach the same low error rates without a latched readout.

The $\left\vert \downarrow \downarrow \right\rangle$ state on the other hand is mapped to the (2, 0) charge state, which is metastable at L (Fig. 1c). *P*_unblocked_ is plotted as a function of *t*_L_ for a prepared $\left\vert \downarrow \downarrow \right\rangle$ state in Fig. 3b. A two-step exponential decay fit to the data reveals an initial decay to a probability of 98.3% due to the imperfect initialization of $\left\vert \downarrow \downarrow \right\rangle$, followed by the main exponential decay due to the metastable (2, 0) charge state relaxing to (1, 0) with ${\Gamma }_{\mathrm{QD}1}^{-1}=206\,\,{\upmu}{\mathrm{s}}$.

This metastable decay time can now limit the readout fidelity of $\left\vert \downarrow \downarrow \right\rangle$ when the measurement time *t*_int_ is not much faster. The (2, 0) readout error can be estimated as the fraction of shots $1-{{\rm{e}}}^{-{\Gamma }_{\mathrm{QD1}}{t}_{\mathrm{int}}/2}$ that have decayed before *t*_int_/2 (single-shot threshold placed midpoint between the two bimodal outcomes). In our case with *t*_int_ = 50 μs, this error would be 11.4%. The double-latching technique allows for the reduction of this error. Waiting for an optimal time *t*_L,opt_ such that ${\Gamma }_{\mathrm{QD}2}^{-1}\ll \,{t}_{\mathrm{L,opt}}\ll {\Gamma }_{\mathrm{QD}1}^{-1}$ at point L before pulsing to DL prevents further decay of the (2, 0) state^10^. This additional lifetime enhancement comes from the detuning dependence of the metastable charge lifetime: near *ε* = 0 (that is, in the PSB window), the interdot tunnel coupling hybridizes the double dot and partially lifts the latching condition^8^. In Fig. 3c, we see that using double latching, we do not observe notable decay (for both $\left\vert \downarrow \downarrow \right\rangle$ and $\left\vert \uparrow \downarrow \right\rangle$) for up to 200 μs. In this case, with *t*_L_ = 2 μs, the (2, 0) decay error during integration is reduced to 0.97%.

## Single-qubit SPAM fidelity

**Fig. 4:**
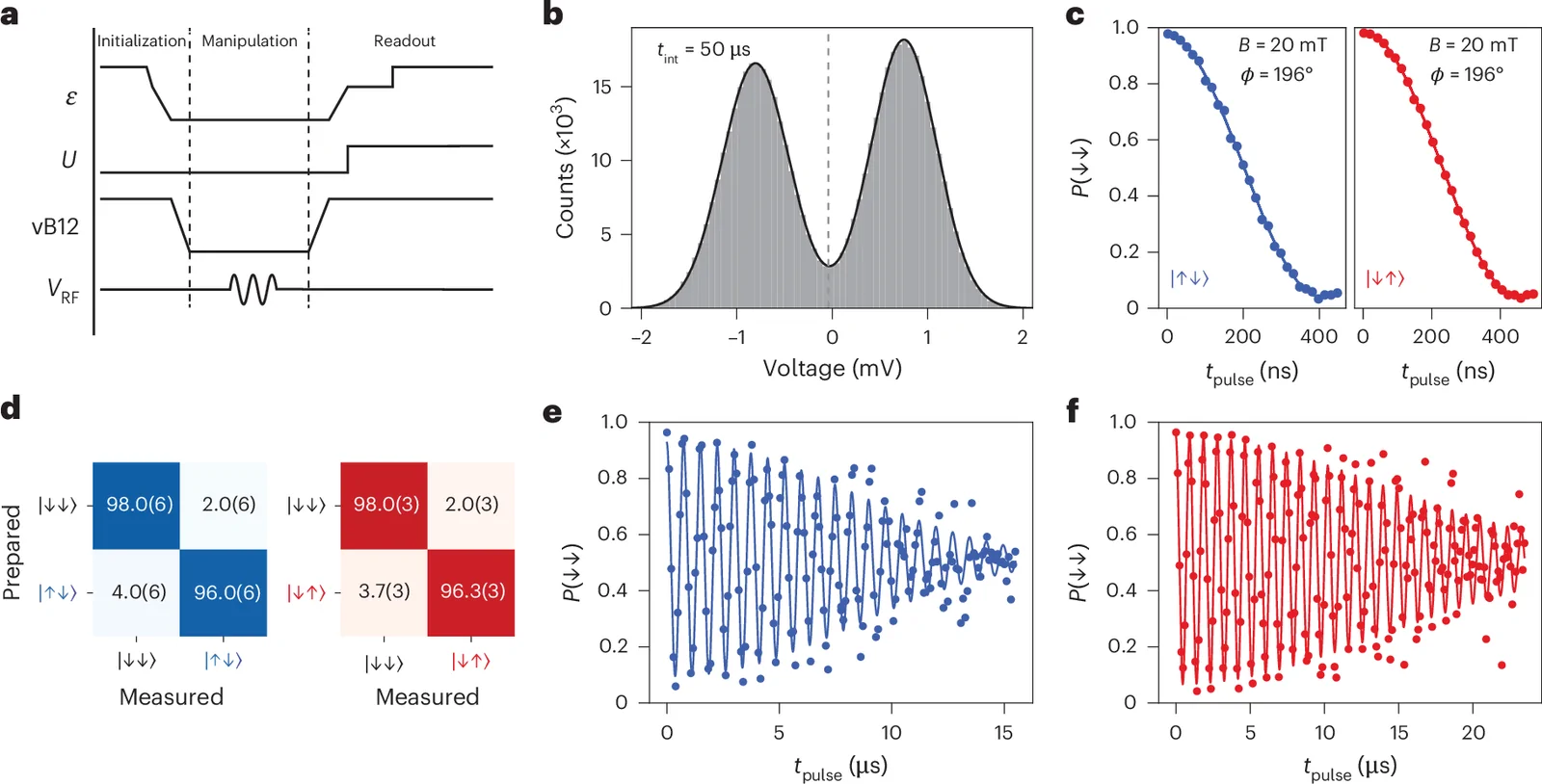
Single-qubit SPAM fidelities. **a**, Complete pulse sequence used in this experiment, separated in abstract steps ‘initialization’, ‘manipulation’ and ‘readout’. **b**, Histogram of all single-shot measurements for the first half-period Rabi oscillation of qubit 1 for an integration time *t*_int_ of 50 μs. The threshold used for each pulse time to obtain single-shot readout fidelities is set to the midway point between the means of two fitted Gaussian distributions. **c**, Detailed Rabi oscillations (pulse time *t*_pulse_) between $\left\vert \downarrow \downarrow \right\rangle$ and $\left\vert \uparrow \downarrow \right\rangle$ (blue) and between $\left\vert \downarrow \downarrow \right\rangle$ and $\left\vert \downarrow \uparrow \right\rangle$ (red) with a fit to extract the SPAM fidelities for each qubit. $P(\left\vert \downarrow \downarrow \right\rangle )$ is the fraction of shots assigned to the $\left\vert \downarrow \downarrow \right\rangle$ state. **d**, The confusion matrices associated with the fits in **c** in percentage. The average SPAM fidelity is 97.0(5)%. **e**,**f**, Rabi oscillations for Q1 (**e**) and Q2 (**f**) and their decay envelope fits, giving Rabi decay times of ${T}_{2}^{{\rm{R}}}=10.8(7)\,\upmu {\rm{s}}$ for **e** and ${T}_{2}^{{\rm{R}}}=19.6(13)\,{\mu} {\mathrm{s}}$ for **f**. The digits in parenthesis represent the 1*σ* confidence interval.

Now that the relevant readout processes and mapping errors have been characterized, we combine this learning to maximize the single-shot SPAM fidelity. The strictest test is to measure the visibility of coherent Rabi oscillations (maximum minus minimum on a probability scale) for each qubit while the other spin is considered as an ancilla. The full pulse sequence is described in Fig. 4a. These measurements are performed at *B* = 20 mT to maximize the decay time at the PSB point. The in-plane field angle is set to *ϕ*_**B**_ = 196° to lower the ramp time required to operate in the super-SAP regime. The transition frequencies of $\left\vert \downarrow \downarrow \right\rangle \leftrightarrow \left\vert \uparrow \downarrow \right\rangle$ (qubit 1) and $\left\vert \downarrow \downarrow \right\rangle \leftrightarrow \left\vert \downarrow \uparrow \right\rangle$ (qubit 2) are 16.61 MHz and 29.24 MHz, respectively. The time at the latch point is set to *t*_L_ = 770 ns. The single-shot $\left\vert \downarrow \downarrow \right\rangle$ probability is assigned by the thresholding of 10,000 shots as described in the ‘State probability determination’ section in Methods. The histogrammed data of all shots obtained up to a half-period Rabi oscillation of qubit 1 is presented in Fig. 4b, with an integration time *t*_int_ of 50 μs, giving an SNR of 2.235.

**Extended Data Fig. 4:**
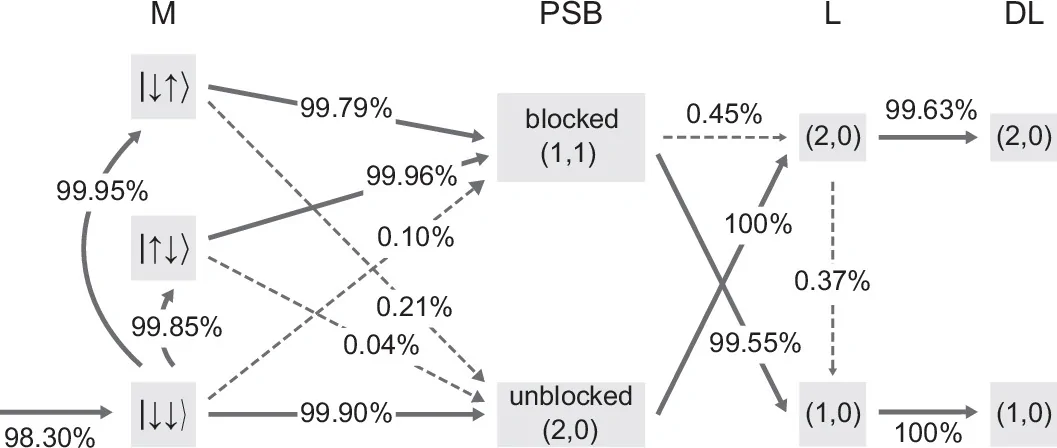
Extracted probabilities of individual readout processes. The sequence is to be interpreted as propagating from left to right.

The SPAM fidelities are reported in Fig. 4d in the form of a confusion matrix. We find an average SPAM fidelity of 97.0(5)% over both qubits, defined as the average of the diagonal elements of the confusion matrix. The visibilities for each qubit are 94.0(9)% and 94.3(4)%. The digits in parenthesis represent the 1*σ* confidence interval throughout this work. We can estimate the contribution of spin-flip errors using the decay envelope of the Rabi oscillations. Rabi decay times ${T}_{2}^{{\rm{R}}}$ of 10.8(7) μs and 19.6(13) μs are obtained by fitting with the function ${{\rm{e}}}^{-{(t/{T}_{2}^{{\rm{R}}})}^{\alpha }}$ with *α* = 2 (Fig. 4e,f). Given a Rabi frequency of 1.1 MHz and 1.2 MHz for each qubit, we estimate spin-flip errors of 0.15% and 0.05% for $\left\vert \uparrow \downarrow \right\rangle$ and $\left\vert \downarrow \uparrow \right\rangle$, respectively. Furthermore, the limited SNR contributes to an error between 1.0% and 1.3% for the total average SPAM fidelity as detailed in the ‘State probability determination’ section in Methods, which could easily be reduced in future work (mostly by increasing the measurement time). A diagram summarizing the probability of each step in the readout scheme is given in the ‘Readout process probabilities’ section in Methods and Extended Data Fig. 4.

## Conclusion

We have reported a step-by-step tune-up of hole spin qubit readout with a SPAM fidelity >97% in Ge using an RF charge sensor and a double-latched readout scheme. The tune-up strategy can also be applied to larger arrays under uniform magnetic field and voltage conditions^32^, as well as to smaller devices^15^, supporting its robustness. Super-SAP is chosen as the StCC regime, because it accommodates the relatively large ${\varDelta }_{{S}{{T}}_{-}}$ and circumvents the challenges of using double ramps that make SAP difficult in this hole system. Super-SAP maps the $\left\vert \downarrow \downarrow \right\rangle$ state to *S*(2, 0) while the other blockaded $\left\vert \uparrow \downarrow \right\rangle ,\left\vert \downarrow \uparrow \right\rangle ,\left\vert \uparrow \uparrow \right\rangle$ states are mapped to (1,1) within the PSB window. The ramp time *t*_ssap_ required to reach the super-SAP regime varies strongly with magnetic field orientation and can be modelled accurately from knowledge of the *g*-tensors and spin-flip tunnelling. We operate at a magnetic field orientation that reduces *t*_ssap_. However, the compatibility of this orientation with qubit control sweetspots requires further investigation.

After StCC, the (1,1) lifetimes are limited by spin decay at the PSB point. We find that these are relatively short (≲20 μs) and can be extended by operating at low magnetic field. This relaxation alone would yield an average readout fidelity of 64% for the PSB readout without latching, assuming the same *t*_int_ = 50 μs. This is the main reason why readout in Ge, and for holes in general, is challenging; it requires either very fast charge detection (*t*_int_ = 1 μs and 25 μs lifetime would give 99% fidelity) or a signal-lifetime-enhancing readout. Further extension of the PSB decay time could be explored in future work, such as by tuning of the tunnel couplings, detuning, orbital spacings, out-of-plane field and so on but might yield only modest improvements. We note that the conditions that lead to a high-fidelity parity-mode readout^9^ could not be reached in our parameter range. The enhanced latching readout (without double latching) increases this fidelity to a predicted value of 92.5% under the same measurement conditions by replacing the fast relaxing (1, 1) spin states with the stable (1, 0) state. This readout fidelity is limited to a small extent by the fork but more so by the metastable nature of the (2, 0) charge state. The lifetime of the metastable state is then itself extended by pulsing to a double-latched point at a higher detuning. With this, we achieve an average single-qubit SPAM fidelity of 97.0(5)%, limited by $\left\vert \downarrow \downarrow \right\rangle$ initialization errors (1.7%) as well as SNR errors (≲1.3%). Both of these error sources could be reduced in future work, and thus, SPAM fidelities above 99% should be possible.

We note that speed of the enhanced latching readout is fundamentally similar to, or better than, the PSB readout: the steps before the final signal integration are usually shorter than the integration time, and the signal is triggered deterministically at the beginning of the integration window because the reservoir tunnelling events are fast, while the signal is typically enhanced by the larger charge response of the sensor. Our work provides a detailed understanding of readout error processes, which we have used here to optimize the single-shot readout fidelity. Moreover, the approach provides increased fidelities and does so while maintaining qubit control, which is a crucial step for the implementation of quantum computing protocols on this platform.

## Methods

### Device and experimental setup

A false-coloured scanning electron microscopy image of the device used in this experiment is presented in Extended Data Fig. 1. The device is fabricated on a Ge/SiGe heterostructure containing a strained Ge quantum well. Ohmic contacts to the quantum well are defined by diffusion of Pt into the upper SiGe barrier at 300 °C. Two gate layers are then defined on the surface of the heterostructure, each consisting of SiO_2_ (7 nm) deposited via plasma-enhanced atomic layer deposition followed by Ti/Pd (20 nm) deposited in a lift-off process together with e-beam lithography. Two hole QDs are formed underneath gates P1 and P2, with the exchange coupling being tuned by gate B12. The RF single-hole transistor is formed underneath S1. The device is packaged in 100-nm SiO_2_ deposited via plasma-enhanced chemical vapour deposition, with the gates being contacted through vias in the oxide with W interconnects to a bondpad layer. A parallel plate capacitor with a capacitance of *C*_p_ ≈ 6 pF is formed between the bondpad and fanout of each device electrode, together with a screening layer metallization deposited in the first gate layer. The details of the matching circuit are given in Supplementary Section 1.

The measurements are conducted in a Bluefors LD400 dilution refrigerator. The sample chip containing the device together with the NbN nanowire inductor chip are both glued onto a QDevil QBoard circuit board. Direct current voltages are applied to the device gates via a QDevil QDAC through direct current looms inside the fridge that are filtered at the millikelvin stage with a QDevil Qfilter. All plunger gates, barrier gates and the inductor connected to the sensor ohmic are attached to on-PCB bias tees that are connected to attenuated coaxial lines for the application of RF signals. The RF signals are generated using Tektronix AWG5024 arbitrary waveform generators.

The ohmic of the charge sensor on the right-hand side of the device is connected to a resonant tank circuit in the form of an NbN superconducting nanowire inductor on a separate chiplet with *L* = 800 nH, leading to a resonance frequency of 72.7 MHz. The resonant excitation signal is applied to the resonator only during *t*_int_ using a Quantum Machines OPX+ (25 mVp) via an attenuated line (−43 dB) and a directional coupler (−20 dB) mounted on the mixing chamber plate, and the reflected signal is amplified by a Cosmic Microwave CMT-BA1 cryogenic amplifier at 4 K (+32 dB), followed by further amplification at room temperature (B&Z 0.1–3 GHz, +37 dB). The readout signal is detected and demodulated by the OPX+. The dilution refrigerator is operated at 500 mK to counteract readout signal shifts attributed to heating of the device that occurs when applying readout pulses to the resonator. This temperature didn’t have a noticeable effect on the measured relaxation times.

An American Magnetics three-axis magnet with a maximum field of (1, 1, 6) T in the (*x*, *y*, *z*) directions was used to apply the magnetic field and a high-stability current source was used for all coils.

### Virtual gates, detuning and on-site energy

Virtual gates are used to compensate the cross-capacitance between different physical gates when tuning the electrochemical potentials of the QDs. These are defined in terms of a virtual gate matrix.

The detuning and on-site energy are defined as linear combinations of virtual gate voltages

B1 $$\begin{array}{r}\left(\begin{array}{r}{\rm{vP1}}\\ {\rm{vP2}}\end{array}\right)=\left(\begin{array}{rc}-0.5&0.5\\ 0.5&0.5\end{array}\right)\left(\begin{array}{r}\varepsilon \\ U\end{array}\right).\end{array}$$

### Measurement of qubit *g*-tensors

The qubit effective *g*-factors are extracted by determining the resonance frequency as a function of magnetic field orientation at *B* = 80 mT, as presented in Extended Data Fig. 2. The *g*-tensor of each qubit is found by a fit to the measured *g*-factors, giving

C1 $${\hat{g}}_{{\rm{Q1}}}=\left(\begin{array}{ccc}0.046&-0.075&-0.038\\ -0.075&0.384&-0.007\\ -0.038&-0.007&12.4\end{array}\right),$$

C2 $${\hat{g}}_{{\rm{Q2}}}=\left(\begin{array}{ccc}0.07&-0.109&0.078\\ -0.109&0.33&0.028\\ 0.078&0.028&12.499\end{array}\right).$$

### PSB decay versus in-plane magnetic field orientation

Extended Data Fig. 3 shows the angular dependence of the decay times inside the PSB window. Decay times are extracted by fitting to an exponential (with offsets). The data are not completely compatible because there is also a detuning dependence to these times, and this was not consistently controlled in this dataset. Nevertheless, we do not find special angles where the decay times become very long or very short. Instead, the decay time varies by about a factor two.

### StCC mapping schemes

Extended Data Table 1 presents a summary of the different StCC maps. Fully defining the qubit readout in the quantum computing sense requires specifying the qubit states, StCC map and singlet–triplet or parity-mode PSB regime.

#### Qubit states

For the SPAM result, we focus on spin-1/2 qubit encoding, with the other spin used as an ancilla. However, in our study of the decay mechanisms, we also investigate the full two-spin Hilbert space to gain insights and preserve generality.

#### StCC map

In this work, we use the super-SAP map, which adiabatically converts the $\left\vert \downarrow \downarrow \right\rangle$ state to a non-blockaded singlet state, while all other three two-spin states remain blockaded.

#### PSB regime: singlet–triplet or parity-mode

Fast decay of the blockaded *T*_0_-like state can lead to a parity-mode readout (2 versus 2), as opposed to singlet–triplet-mode readout (1 versus 3).

For all of these steps, the qubit encodings, StCC map and PSB mode can be mixed and matched for achieving a certain result. One must therefore clearly specify the combination used in a given experiment.

After one measurement, and for all the variations, only a single bit of information is acquired, while two bits would be required to fully determine the two-spin state before measurement. One must therefore encode the qubit in a subspace or repeat quasi-non-demolition measurements in different bases.

### State probability determination

The $\left\vert \downarrow \downarrow \right\rangle$ single-shot probability is assigned by the thresholding of 10,000 shots. For each measurement, the in-phase and quadrature demodulated signals are projected along one dimension *V*_1D_ using principal component analysis, followed by a fit of a bimodal Gaussian distribution to the histogrammed data

F1 $$C({V}_{\mathrm{1D}})={A}_{1}{{\rm{e}}}^{\displaystyle\frac{{\left({V}_{\mathrm{1D}}-{\upmu }_{1}-{V}_{\mathrm{off}}\right)}^{2}}{2{\sigma }_{1}^{2}}}+{A}_{2}{{\rm{e}}}^{\displaystyle\frac{{\left({V}_{\mathrm{1D}}-{\upmu }_{2}-{V}_{\mathrm{off}}\right)}^{2}}{2{\sigma }_{2}^{2}}},$$

where *C* is the number of shots for a measured *V*_1D_, and *V*_off_ is an offset of the bimodal distribution from *V*_1D_ = 0 mV. The means μ_*i*_ of each Gaussian *i* are such that μ_1_ < μ_2_. *A*_*i*_ and *σ*_*i*_ are the weight and standard deviation of each Gaussian, respectively. We define the SNR as

F2 $${\rm{SNR}}=\frac{| {\upmu }_{2}-{\upmu }_{1}| }{{\sigma }_{1}+{\sigma }_{2}}.$$

The threshold voltage *V*_th_ for single-shot probability assignment is defined as the midway point between the two means, shifted by *V*_off_

F3 $${V}_{{\rm{th}}}={V}_{{\rm{off}}}+\frac{1}{2}({\upmu }_{2}-{\upmu }_{1}).$$

This is valid for *σ*_1_ = *σ*_2_, which is the case here. All shots with *V*_1D_ < *V*_th_ were assigned as $\left\vert \downarrow \downarrow \right\rangle$.

It is important to distinguish the $\left\vert \downarrow \downarrow \right\rangle$ single-shot probability $P(\left\vert \downarrow \downarrow \right\rangle )$ from the averaged assignment probability $A(\left\vert \downarrow \downarrow \right\rangle )$, which compares the relative areas of each Gaussian. This is given by (*σ*_1_ = *σ*_2_)

F4 $$A(\left\vert \downarrow \downarrow \right\rangle )=\frac{{A}_{1}}{{A}_{1}+{A}_{2}}.$$

$A(\left\vert \downarrow \downarrow \right\rangle )$ allows for the assignment of the $\left\vert \downarrow \downarrow \right\rangle$ probability once the full bimodal Gaussian distribution is known. This distinguishes readout mapping errors from SNR errors that occur during single-shot thresholding. Calculating the total average assignment fidelity for the measurements of Fig. 4 gives 98.0(5)%. This allows us to assign a ~1% error in the measured single-shot SPAM fidelity that arises due to SNR limitations. Alternatively, the SNR error *r*_SNR_ can be calculated as

F5 $${r}_{{\rm{SNR}}}=\frac{1}{2}\mathrm{erfc}\,\left(\frac{{\rm{SNR}}}{\sqrt{2}}\right),$$

where $\mathrm{erfc}\,(x)$ is the complementary error function^33^. For the SNR of 2.235 in this experiment, the calculated *r*_SNR_ is 1.27%.

### Readout process probabilities

The probabilities of each step occurring in the double-latched readout scheme are summarized in Extended Data Fig. 4. The StCC errors (dashed arrows between M and PSB) are calculated as $1-\sqrt{1-{r}_{{\rm{StCC}}}}$ for the *r*_StCC_ of each state as presented in the main text.

## Supplementary information

Supplementary Information Supplementary Sections 1–3 and Fig. 1.

## Data Availability

The data that support the findings of this study are available via Zenodo at https://doi.org/10.5281/zenodo.21671988 (ref. ^34^).

## Extended data

**Extended Data Table 1:** Different StCC schemes for mapping of spin states at point M to spin states at the PSB point

	super-SAP	SAP	RAP
|↑↑〉	|S̃(1,1)〉	|T_+_(1,1)〉	|T_+_(1,1)〉
|↓↑〉	|T̃_0_(1,1)〉	|T̃_0_(1,1)〉	α|S̃(2,0)〉 + β|T̃_0_(1,1)〉
|↑↓〉	|T₋(1,1)〉	|S(2,0)〉	β*|S̃(2,0)〉 − α*|T̃_0_(1,1)〉
|↓↓〉	|S(2,0)〉	|T₋(1,1)〉	|T₋(1,1)〉

Here *α* and *β* are complex probability amplitudes with |*α|*^2^ + |*β*|^2^ = 1.

## References

[1] Lodari, M. et al. Low percolation density and charge noise with holes in germanium. *Mater. Quantum. Technol.* **1**, 011002 10.1088/2633-4356/abcd82 (2021).

[2] Massai, L. et al. Impact of interface traps on charge noise and low-density transport properties in Ge/SiGe heterostructures. *Comm. Mater.* **5**, 151 10.1038/s43246-024-00563-8 (2024). PMC11324522 39157449

[3] Hendrickx, N. W. et al. A single-hole spin qubit. *Nat. Comm.* **11**, 3478 10.1038/s41467-020-17211-7 (2020). PMC7351715 32651363

[4] Hendrickx, N. W. et al. Sweet-spot operation of a germanium hole spin qubit with highly anisotropic noise sensitivity. *Nat. Mater.* **23**, 920–927 10.1038/s41563-024-01857-5 (2024). PMC11230914 38760518

[5] Wang, C.-A. et al. Operating semiconductor quantum processors with hopping spins. *Science* **385**, 447 10.1126/science.ado5915 (2024). 39052794

[6] Lawrie, W. I. et al. Simultaneous single-qubit driving of semiconductor spin qubits at the fault-tolerant threshold. *Nat. Comm.* **14**, 3617 10.1038/s41467-023-39334-3 (2023). PMC10279658 37336892

[7] Vigneau, F. et al. Probing quantum devices with radio-frequency reflectometry. *Appl. Phys. Rev.* **10**, 021305 10.1063/5.0088229 (2023).

[8] Harvey-Collard, P. et al. High-fidelity single-shot readout for a spin qubit via an enhanced latching mechanism. *Phys. Rev. X* **8**, 021046 10.1103/PhysRevX.8.021046 (2018).

[9] Hendrickx, N. W. et al. A four-qubit germanium quantum processor. *Nature* **591**, 580 10.1038/s41586-021-03332-6 (2021). 33762771

[10] Kiyama, H., van Hien, D., Ludwig, A., D. Wieck, A. & Oiwa, A. High-fidelity spin readout via the double latching mechanism. *npj Quantum Inf.* **10**, 95 10.1038/s41534-024-00882-1 (2024).

[11] Huang, J. Y. et al. High-fidelity spin qubit operation and algorithmic initialization above 1 K. *Nature* **627**, 772–777 10.1038/s41586-024-07160-2 (2024). PMC10972758 38538941

[12] Philips, S. G. J. et al. Universal control of a six-qubit quantum processor in silicon. *Nature* **609**, 919 10.1038/s41586-022-05117-x (2022). PMC9519456 36171383

[13] Blumoff, J. Z. et al. Fast and high-fidelity state preparation and measurement in triple-quantum-dot spin qubits. *PRX Quantum* **3**, 010352 10.1103/PRXQuantum.3.010352 (2022).

[14] Takeda, K. et al. Rapid single-shot parity spin readout in a silicon double quantum dot with fidelity exceeding 99%. *npj Quantum Inf.* **10**, 22 10.1038/s41534-024-00813-0 (2024).

[15] Tsoukalas, K. et al. A dressed singlet–triplet qubit in germanium. *Nat. Commun.* **17**, 699 10.1038/s41467-025-65569-3 (2026). PMC12820228 41559029

[16] Kelly, E. G. et al. Capacitive crosstalk in gate-based dispersive sensing of spin qubits. *Appl. Phys. Lett.* **123**, 262104 10.1063/5.0177857 (2023).

[17] Eggli, R. S. et al. Coupling a high-Q resonator to a spin qubit with all-electrical control. *Phys. Rev. Res.* **7**, 013197 10.1103/PhysRevResearch.7.013197 (2025).

[18] Taylor, J. M. et al. Relaxation, dephasing, and quantum control of electron spins in double quantum dots. *Phys. Rev. B* **76**, 035315 10.1103/PhysRevB.76.035315 (2007).

[19] Danon, J. & Nazarov, Y. V. Pauli spin blockade in the presence of strong spin-orbit coupling. *Phys. Rev. B* **80**, 041301 10.1103/PhysRevB.80.041301 (2009).

[20] Studenikin, S. A. et al. Enhanced charge detection of spin qubit readout via an intermediate state. *Appl. Phys. Lett.* **101**, 233101 10.1063/1.4749281 (2012).

[21] Jirovec, D. et al. Dynamics of hole singlet–triplet qubits with large g-factor differences. *Phys. Rev. Lett.* **128**, 126803 10.1103/PhysRevLett.128.126803 (2022). 35394319

[22] Petta, J. R. et al. Coherent manipulation of coupled electron spins in semiconductor quantum dots. *Science* **309**, 2180 10.1126/science.1116955 (2005). 16141370

[23] Harvey-Collard, P. et al. All-electrical universal control of a double quantum dot qubit in silicon MOS, in *2017 IEEE International Electron Devices Meeting (IEDM)* 36.5.1–36.5.4 10.1109/iedm.2017.8268507 (IEEE, 2017).

[24] Nichol, J. M. et al. Quenching of dynamic nuclear polarization by spin-orbit coupling in GaAs quantum dots. *Nat. Commun.* **6**, 7682 10.1038/ncomms8682 (2015). PMC4518271 26184854

[25] Geyer, S. et al. Anisotropic exchange interaction of two hole–spin qubits. *Nat. Phys.* **20**, 1152, 10.1038/s41567-024-02481-5 (2024). PMC11631753 39664598

[26] Saez-Mollejo, J. et al. Exchange anisotropies in microwave-driven singlet–triplet qubits. *Nat. Commun.* **16**, 3862 10.1038/s41467-025-58969-y (2025). PMC12022185 40274808

[27] Nurizzo, M. et al. Complete readout of two-electron spin states in a double quantum dot. *PRX Quantum* **4**, 010329 10.1103/PRXQuantum.4.010329 (2023).

[28] Watson, T. F. et al. Atomically engineered electron spin lifetimes of 30 s in silicon. *Sci. Adv.* **3**, e1602811 10.1126/sciadv.1602811 (2017). PMC5477090 29159289

[29] Camenzind, L. C. et al. Hyperfine-phonon spin relaxation in a single-electron GaAs quantum dot. *Nat. Commun.* **9**, 3454 10.1038/s41467-018-05879-x (2018). PMC6110844 30150721

[30] Kornich, V., Kloeffel, C. & Loss, D. Phonon-assisted relaxation and decoherence of singlet–triplet qubits in Si/SiGe quantum dots. *Quantum* **2**, 70 10.22331/q-2018-05-28-70 (2018).

[31] Seedhouse, A. E. et al. Pauli blockade in silicon quantum dots with spin-orbit control. *PRX Quantum* **2**, 010303 10.1103/PRXQuantum.2.010303 (2021).

[32] Seidler, I. et al. Spatial uniformity of g-tensor and spin-orbit interaction in germanium hole spin qubits. Preprint at https://arxiv.org/abs/2510.03125 (2025).

[33] Krantz, P. et al. A quantum engineer’s guide to superconducting qubits. *Appl. Phys. Rev.* **6**, 021318 10.1063/1.5089550 (2019).

[34] Eoin Gerard, K. Identification and mitigation of errors in hole spin qubit readout—open access data. *Zenodo*10.5281/zenodo.21671988 (2026).

