# Supplementary material for: Identification and mitigation of errors in hole spin qubit readout
Source: Nat Electron. 2026 Sep 29;9(10):1089–97. doi: 10.1038/s41928-026-01705-1 (PMC13690583; doi:10.1038/s41928-026-01705-1)
Supplement: Supplementary file 1 [file 41928_2026_1705_MOESM1_ESM.pdf]

nature electronics

Supplementary information https://doi.org/10.1038/s41928-026-01705-1

Identification and mitigation of errors in hole spin qubit readout

In the format provided by the authors and unedited

Supplementary information for: Identification and mitigation of errors in hole spin qubit readout

Supplementary Section 1: RF matching circuit

The simplified matching circuit used in this work is presented in Fig. Ext. Data 1. A resonant RF signal is coupled into the tank circuit via a coupling capacitor $C_\mathrm{c} = 10\,\mathrm{pF}$. A spurious matching capacitance of $C_\mathrm{m} = 5\,\mathrm{pF}$ is necessary to include in the simulation to account for PCB parasitics. The sensor ohmic connected to the inductor is biased through a bias V_S applied through a $R_\mathrm{BT} = 1\,\mathrm{M\Omega}$ resistor. The parasitic capacitance C_p comes mainly from the parallel plate capacitance between the ohmic bondpad plus fanout and the screening layer below them (the purpose of which was discussed in the main text). The sensor drain ohmic is biased with a voltage V_D. All experiments reported in the main text were performed with $V_\mathrm{S} = V_\mathrm{D} = 0$.

To optimize the charge sensitivity of the matching circuit as a function of different circuit parameters, we defined a measure of sensitivity $|\Delta S|$ as the change in reflected signal when the (sensor) load resistance changes from $100\,\mathrm{k\Omega}$ to $1\,\mathrm{M\Omega}$. This estimates a realistic change in impedance of a charge sensor in the Coulomb blockade regime. We then numerically simulate for $|\Delta S|$ as a function of C_p vs. L and C_p vs. C_m (Fig. S1). The white points show the actual parameters used in this work. Reducing the parasitic capacitance C_p to the screening layer, increasing L and also using a higher value of C_m would increase the sensitivity of the RF charge sensor leading to a higher signal-to-noise ratio. However, increasing LC_m is not feasible due to the reduction in the resonance frequency that would then become attenuated by high-pass filters on the lines, and C_p has already been minimised to the extent allowed by the screening layer. Hence our parameters are an optimum given these constraints.

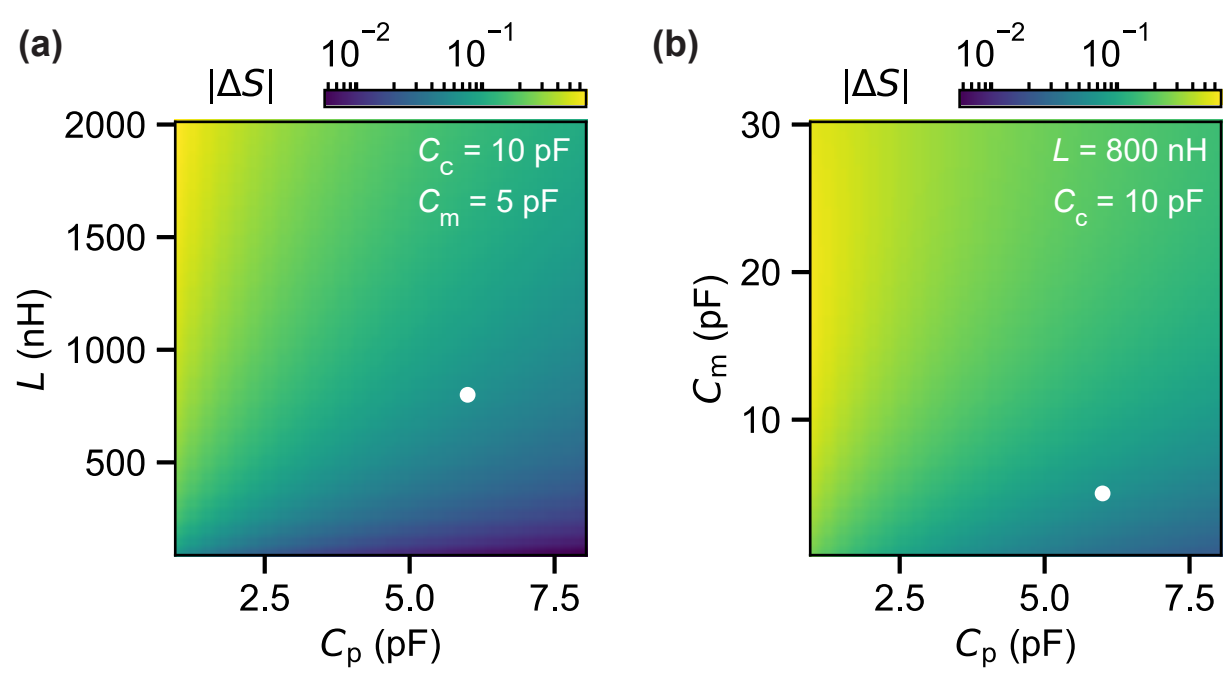

Supplementary Fig 1. Numerical simulations of S-parameter difference $|\Delta S|$ for different circuit parameters. **(a)** C_p vs L and **(b)** C_p vs C_m. The white points indicate the actual circuit parameters used in this work.

Supplementary Section 2: Simulation of the ramp rates

The single-particle Hamiltonian of a double QD with spin-orbit interaction reads [25]

$$\begin{aligned} H_{1P} = &-\frac{\varepsilon}{2}(|1\rangle\langle 1| - |2\rangle\langle 2|) \\ &+ \frac{\mu_\mathrm{B}}{2}\mathbf{B}\cdot(|1\rangle\langle 1|\,\hat{g}_{\mathrm{Q1}} + |2\rangle\langle 2|\,\hat{g}_{\mathrm{Q2}})\boldsymbol{\sigma} \\ &+ [t_\mathrm{o}\,|1\rangle\langle 2| - it_\mathrm{so}\mathbf{n}_\mathrm{so}\cdot\boldsymbol{\sigma}\,|1\rangle\langle 2| + h.c.], \end{aligned} \tag{2.1}$$

where $|i\rangle$ is the first orbital state of QDi, $\boldsymbol{\sigma}$ is the spin vector operator with $\sigma_z = |{\uparrow}\rangle\langle{\uparrow}| - |{\downarrow}\rangle\langle{\downarrow}|$, t_o (t_so) is the spin-conserving (spin-flip) tunnel-coupling and $h.c.$ denotes the Hermitian conjugate. Here ε is the detuning in terms of energy and not voltage as in the main text.

Using the single-particle Hamiltonian from Eq. (2.1) we construct a particle-exchange-symmetric two-particle Hamiltonian

$$H_{2P} = H_{1P}\otimes\mathbb{1} + \mathbb{1}\otimes H_{1P} + U_H(|1\rangle\langle 1|\otimes|1\rangle\langle 1| + |2\rangle\langle 2|\otimes|2\rangle\langle 2|), \tag{2.2}$$

where U_H is the Hubbard charging energy (not the virtual control parameter). Since the eigenvalues corresponding to particle-exchange-antisymmetric wavefunctions of Eq. (2.2) obey Pauli's exclusion principle, we project the symmetric Hamiltonian to the following antisymmetric basis states

$$|S(2{,}0)\rangle_0 = \frac{1}{\sqrt{2}}(|1\uparrow\rangle \otimes |1\downarrow\rangle - |1\downarrow\rangle \otimes |1\uparrow\rangle), \tag{2.3a}$$

$$|ss'\rangle_0 = \frac{1}{\sqrt{2}}(|1s\rangle \otimes |2s'\rangle - |2s'\rangle \otimes |1s\rangle), \tag{2.3b}$$

where we used the 0 subscript to distinguish the basis states from the eigenstates at the manipulation point ε_M. The projection leads to the low-energy Hamiltonian

$$H_{5\times5} = \begin{pmatrix} U_H - \varepsilon & t_\mathrm{so}(n^y_\mathrm{so} - i n^x_\mathrm{so}) & t_\mathrm{o} + i t_\mathrm{so} n^z_\mathrm{so} & -t_\mathrm{o} + i t_\mathrm{so} n^z_\mathrm{so} & t_\mathrm{so}(n^y_\mathrm{so} + i n^x_\mathrm{so}) \\ t_\mathrm{so}(n^y_\mathrm{so} + i n^x_\mathrm{so}) & \Delta^z_1 + \Delta^z_2 & \Delta^x_2 - i\Delta^y_2 & \Delta^x_1 - i\Delta^y_1 & 0 \\ t_\mathrm{o} - i t_\mathrm{so} n^z_\mathrm{so} & \Delta^x_2 + i\Delta^y_2 & \Delta^z_1 - \Delta^z_2 & 0 & \Delta^x_1 - i\Delta^y_1 \\ -t_\mathrm{o} - i t_\mathrm{so} n^z_\mathrm{so} & \Delta^x_1 + i\Delta^y_1 & 0 & -\Delta^z_1 + \Delta^z_2 & \Delta^x_2 - i\Delta^y_2 \\ t_\mathrm{so}(n^y_\mathrm{so} - i n^x_\mathrm{so}) & 0 & \Delta^x_1 + i\Delta^y_1 & \Delta^x_2 + i\Delta^y_2 & -\Delta^z_1 - \Delta^z_2 \end{pmatrix}, \tag{2.4}$$

where the order of the basis states is $\{|S(2,0)\rangle_0, |\uparrow\uparrow\rangle_0, |\uparrow\downarrow\rangle_0, |\downarrow\uparrow\rangle_0, |\downarrow\downarrow\rangle_0\}$ and we defined the Zeeman vectors as $\boldsymbol{\Delta}_i = \frac{1}{2}\mu_\mathrm{B}\mathbf{B}\cdot\hat{g}_{\mathrm{Q}i}$.

To simulate the experiment in Fig. 2(c) we assume that the ramp in was perfectly adiabatic, i.e., the starting state is a pure state $|\downarrow\downarrow\rangle$, which is the lowest-energy eigenstate of $H_{5\times5}(\varepsilon_\mathrm{M})$. Note that this assumption neglects the thermalization effect during the ramp in with the $|\uparrow\downarrow\rangle$ state, which would reduce the contrast in the experiment. After the ramp out, the measurement probability assigned to the $|\downarrow\downarrow\rangle$ state is calculated as

$$P(\downarrow\downarrow) = |\langle S(2,0)|_0\, U(t_\mathrm{ramp})\, |\downarrow\downarrow\rangle|^2, \tag{2.5}$$

where we used $|S(2,0)\rangle_0$ as a measurement projector, and note that using another close-by state that is more appropriate for the description of the latching would only reduce the range of $P(\downarrow\downarrow)$, i.e., the contrast. The propagator in Eq. (2.5) is

$$U(t) = \mathcal{T} e^{-i\int_0^t dt' H_{5\times5}(\varepsilon(t'))/\hbar}, \tag{2.6}$$

with $\varepsilon(t) = \varepsilon_M + (\varepsilon_\mathrm{PSB} - \varepsilon_M)t/t_\mathrm{ramp}$ and where $\mathcal{T}$ denotes the time ordered integral.

The tunnel couplings $t_\mathrm{o}/h = 0.79\,\mathrm{GHz}$ and $t_\mathrm{so}/h = 0.125\,\mathrm{GHz}$ and spin-orbit vector $\mathbf{n}_\mathrm{so} = (0.81, 0.59, 0)$ were found by generating map plots like the experimental data of Fig. 2(c) for different parameter sets, extracting the lines of the characteristic ramp rate given by $P(\downarrow\downarrow) = 1/e$, and performing a least squares minimization to the corresponding lines of the measured data. In this fitting process we disregard the $\phi_\mathbf{B} = 90°$ data from Fig. 2(c) due to the absence of corresponding g-factor data. This way, we found significantly better agreement with the measured data and an in-plane spin-orbit vector. Including the data from the $\phi_\mathbf{B} = 90°$ plane strongly violates this expected symmetry.

Finally, the ramp rate used in the Landau-Zener formula is defined as:

$$\frac{\mathrm{d}E_{\mathrm{ST}_-}}{\mathrm{d}\varepsilon} = \frac{E_{\mathrm{ST}_-}(\varepsilon_\mathrm{ax}) - E_{\mathrm{ST}_-}(\varepsilon_\mathrm{ax} - 3\Delta_\mathrm{ST})}{3\Delta_\mathrm{ST}} \tag{2.7}$$

where ε_ax is the detuning where the ST_- gap is minimal. We determined the range of validity for this formula as $E_{\mathrm{ST}_-}(\varepsilon_\mathrm{M}) > E_{\mathrm{ST}_-}(\varepsilon_\mathrm{ax} - 4\Delta_\mathrm{ST})$, which ensures that there is a wide enough dip in the ST_- gap such that the transition can be treated as an anticrossing of two energy levels.

Supplementary Section 3: Theoretical model of the energy spectrum

In order to account for the finite size of the spin blockade window (i.e., the high energy (2,0) triplet states, as shown in Fig. 1(b)), we extend the single-particle Hamiltonian in Eq. (2.1) with an extra orbital state $|1'\rangle$ on QD1. The modified single-particle Hamiltonian reads

$$\begin{aligned} H_{1P'} = H_{1P} &+ \left(-\frac{\varepsilon}{2} + \Delta_\mathrm{orb}\right)|1'\rangle\langle 1'| \\ &+ \frac{\mu_\mathrm{B}}{2}\mathbf{B}\cdot\hat{g}_{\mathrm{Q}1'}\boldsymbol{\sigma}\,|1'\rangle\langle 1'| \\ &+ [t'_\mathrm{o}\,|1'\rangle\langle 2| - i t'_\mathrm{so}\mathbf{n}'_\mathrm{so}\cdot\boldsymbol{\sigma}\,|1'\rangle\langle 2| + h.c.], \end{aligned} \tag{3.1}$$

where we define the orbital splitting in QD1 as $\Delta_{\rm orb}$, as well as orbital-specific g-tensor, tunnel couplings and spin-orbit vector. This specificity is necessary in full generality to account for the orbital dependence of the tunnel couplings, g-tensors, and so on. In the energy spectrum shown in the main text however, we do not distinguish between the $|1\rangle$ and $|1'\rangle$ orbitals in terms of these parameters, as many of these differences (apart from $\Delta_{\rm orb}$ which is simply the size of the PSB window) are not known quantitatively.

Using the single-particle Hamiltonian from Eq. (3.1) we construct a particle-exchange-symmetric two-particle Hamiltonian

$$\begin{aligned} H_{2P'} &= H_{1P'} \otimes \mathbb{1} + \mathbb{1} \otimes H_{1P'} \\ &\quad + U_H \sum_{i,j \in \{1,1'\}} \left(|i\rangle\langle i| \otimes |j\rangle\langle j| + |2\rangle\langle 2| \otimes |2\rangle\langle 2| \right), \end{aligned} \tag{3.2}$$

where U_H is the Hubbard charging energy. To project this Hamiltonian to the physical subspace, in addition to the five states in Eq. (2.3a)-(2.3b) we consider three triplet-like basis states:

$$|T_{ss}(2{,}0)\rangle_0 = \frac{1}{\sqrt{2}}(|1s\rangle\,|1's\rangle - |1's\rangle\,|1s\rangle), \tag{3.3a}$$

$$|T_0(2{,}0)\rangle_0 = \frac{1}{2}(|1\uparrow\rangle\,|1'\downarrow\rangle + |1\downarrow\rangle\,|1'\uparrow\rangle - |1'\uparrow\rangle\,|1\downarrow\rangle - |1'\downarrow\rangle\,|1\uparrow\rangle). \tag{3.3b}$$

We do not consider the singlet-like state of the 1-1′ orbitals because it is even higher in energy due to the single-dot exchange energy.
